# The association between Dietary Index for Gut Microbiota and sarcopenia: the mediating role of Dietary Inflammatory Index

**DOI:** 10.3389/fnut.2025.1514209

**Published:** 2025-03-31

**Authors:** Hongyang Gong, Shuqin Duan, Xiaomei Lin, Shaoqun Huang

**Affiliations:** ^1^Department of Oncology Surgery, Fuzhou Hospital of Traditional Chinese Medicine Affiliated to Fujian University of Traditional Chinese Medicine, Fuzhou, Fujian, China; ^2^Department of Physiology, College of Medicine, Chosun University, Gwangju, Republic of Korea; ^3^Department of Obstetrics and Gynaecology, The Second Hospital of Jilin University, Changchun, Jilin, China; ^4^Department of Orthopedics, Fuzhou Hospital of Traditional Chinese Medicine Affiliated to Fujian University of Traditional Chinese Medicine, Fuzhou, Fujian, China

**Keywords:** Dietary Index for Gut Microbiota, Dietary Inflammatory Index, sarcopenia, NHANES, mediation analysis

## Abstract

**Background:**

Given the global changes in environmental and dietary habits, understanding the potential impact of dietary factors and diet-related inflammation on skeletal muscle diseases, including sarcopenia, is crucial. Investigating these relationships can aid in the development of more effective prevention strategies. This study used the Dietary Index for Gut Microbiota (DI-GM) and the Dietary Inflammatory Index (DII) as diet-related variables. DI-GM is a scoring system used to assess the influence of diet on Gut Microbiota health. Additionally, DII quantifies the inflammatory potential of a diet. This study explores the association between DI-GM and sarcopenia and evaluates whether DII moderates this relationship.

**Methods:**

This study conducted a cross-sectional analysis of 9,470 participants from the 2011–2018 NHANES database. Multivariable logistic regression, restricted cubic splines (RCS), and subgroup analysis were employed to examine the association between DI-GM and the prevalence of sarcopenia. Additionally, mediation analysis was performed to investigate the potential associations between DII, DI-GM, and sarcopenia.

**Results:**

A total of 9,470 participants were included in this study, of whom 823 (7%) had sarcopenia. After adjusting for all variables using multivariable logistic regression, each one-unit increase in DI-GM was associated with a 15% decrease in sarcopenia prevalence (OR = 0.85, 95% CI: 0.77, 0.94), while each one-unit increase in DII was associated with a 28% increase in sarcopenia prevalence (OR = 1.28, 95% CI: 1.17, 1.41). Furthermore, the results remained robust when DI-GM and DII were divided into tertiles. RCS analysis revealed a significant linear relationship between DI-GM and sarcopenia. The results of the subgroup analysis also showed that the above relationships were robust. Mediation analysis showed that 55% of the association between DI-GM and sarcopenia was mediated by DII (*P* < 0.001).

**Conclusion:**

Adhering to dietary recommendations based on DI-GM may reduce the prevalence of sarcopenia. Additionally, DII appears to mediate this relationship, suggesting that an anti-inflammatory diet could offer potential benefits.

## Introduction

Sarcopenia is defined as a progressive and systemic skeletal muscle disease characterized by the loss of muscle mass and function (1). Studies have shown that its prevalence reaches 5–13% among individuals aged 60–70 and rises to 11–50% in those aged 80 and older, posing a significant challenge to healthcare costs. In 2000, the direct medical costs associated with sarcopenia in the United States were estimated at $18.5 billion. A 10% reduction in sarcopenia prevalence could save the U.S. healthcare system $1.1 billion annually (2, 3). Beyond its association with increased mortality risk, sarcopenia has been identified as a critical prognostic indicator for survival and clinical complications in patients with cancer, kidney dysfunction, liver disease, and metabolic disorders (4). Preventing sarcopenia has been a key area of focus, with research identifying age, physical inactivity, metabolic imbalances, and neuromuscular dysfunction as major risk factors (1, 5). Regarding lifestyle factors, numerous studies have explored muscle outcomes related to dietary patterns, suggesting that physical activity and nutritional status, influenced by dietary intake or supplementation, are linked to sarcopenia risk (2, 4).

The composition of the gut microbiota is associated with various aspects of human health, with diet serving as a key determinant of the structure and function of gut microbial communities (6, 7). Research has indicated that dysbiosis can lead to significant alterations in skeletal muscle metabolism through the “gut-muscle axis” and the subsequent loss of microbiota-derived metabolites (8). An animal study suggested that probiotics may regulate age-related muscle damage, and other studies have highlighted that different gut microbial taxa may have varying effects on muscle (9, 10). Recently, a novel DI-GM was proposed (11). Unlike existing indices, DI-GM incorporates specific foods rather than food groups and includes fermented dairy products, which are a unique dietary component critical for gut microbiota health (11).

The Dietary Inflammatory Index (DII) was introduced in 2014 to quantify the effect of dietary components on six inflammatory biomarkers (IL-1β, IL-4, IL-6, IL-10, TNF-α, and C-reactive protein) (12). Chronic inflammation is linked to the development of several non-communicable chronic diseases, and diet may influence these diseases through mechanisms such as gut microbiota regulation (13). One study investigating the relationship between DII and musculoskeletal issues in adults found that pro-inflammatory diets may be associated with such problems (14). This could be due to elevated levels of serum inflammatory cytokines, which are linked to lower muscle mass and poorer muscle function (15). Inflammation may increase the risk of sarcopenia by activating the ubiquitin-proteasome system, enhancing oxidative stress and NF-κB activity, and impairing skeletal muscle protein synthesis (16–18). However, despite the growing research on DI-GM and DII, systematic studies exploring the association between DI-GM, DII, and sarcopenia remain scarce. Investigating these potential associations could not only offer new directions for sarcopenia prevention but also promote the adoption of healthier and more sustainable diets.

This study aims to investigate the association between DI-GM and sarcopenia, with a specific focus on the potential mediating role of DII in this relationship. Given the growing evidence suggesting that diet influences sarcopenia both directly and indirectly through its impact on gut microbiota and systemic inflammation, we hypothesize that DI-GM is inversely associated with sarcopenia and that DII partially mediates this association. To test these hypotheses, we utilized data from the 2005 to 2018 National Health and Nutrition Examination Survey (NHANES), a nationally representative database, to explore these relationships in a large sample.

## Materials and methods

### Study participants

This cross-sectional study utilized data from the National Health and Nutrition Examination Survey (NHANES) conducted by the National Center for Health Statistics (NCHS). The survey aims to collect demographic information regarding the health and nutritional intake of U.S. citizens. The NCHS Research Ethics Review Board approved all NHANES study protocols, and written informed consent was obtained from all participants. Our secondary analysis adhered to the STROBE guidelines for cross-sectional studies and did not require additional Institutional Review Board approval. For more details on NHANES methods and ethical considerations, please refer to the Centers for Disease Control and Prevention (CDC) and the National Health and Nutrition Examination Survey website: https://www.cdc.gov/nchs/nhanes/index.htm.

This cross-sectional study utilized nationally representative data from the National Health and Nutrition Examination Survey (NHANES). Among the 39,156 participants across four NHANES cycles from 2011 to 2018, 16,539 individuals aged 20 years and older and not pregnant were identified. Participants with incomplete data for the Dietary Gut Microbiota Index (DI-GM) and Dietary Inflammatory Index (DII) (*n* = 4,307) were excluded, as were those with incomplete data on sarcopenia (*n* = 8,840). Ultimately, 9,470 participants were included in the study (Supplementary Figure S1).

### Sarcopenia assessment

Total appendicular lean mass (ALM) was assessed using dual-energy X-ray absorptiometry (DEXA) in the NHANES program. Sarcopenia was diagnosed using the Sarcopenia Index, calculated by dividing the total mass of appendicular skeletal muscle (in kg/m^2^) by body mass index (BMI, in kg/m^2^) (19). According to the guidelines from the Foundation for the National Institutes of Health (FNIH), cut-off values for the Sarcopenia Index were set at < 0.789 for men and < 0.512 for women to diagnose sarcopenia (20).

### Definition of DII

The Dietary Inflammatory Index (DII) serves as a key metric for assessing the inflammatory potential of various food components, including vitamins and minerals. A DII score of ≥ 0 indicates a pro-inflammatory diet, while a score of < 0 reflects an anti-inflammatory diet (21). Furthermore, higher DII scores suggest harmful dietary habits, whereas lower DII scores are indicative of healthier dietary practices (22). Supplementary material include a detailed algorithm for calculating the DII score.

### Dietary Index for Gut Microbiota

According to Kase et al. (11), the Dietary Index for Gut Microbiota (DI-GM) comprises 14 food items or nutrients. Beneficial components include fermented dairy products, chickpeas, soy, whole grains, fiber, cranberries, avocados, broccoli, coffee, and green tea, while adverse components consist of red meat, processed meats, refined grains, and diets high in fat (≥40% of energy from fat). Each component is scored as 0 or 1 based on gender-specific median intake levels, and the scores are summed to derive the overall DI-GM score. The specific calculation method for DI-GM is detailed in Supplementary Table S1.

### Covariables

The covariates for this study included age, sex, education level, marital status, poverty-income ratio (PIR), race, smoking, alcohol consumption, physical activity, hypertension, diabetes, and hyperlipidemia. Detailed information regarding these covariates can be found in Supplementary Table S2.

### Statistical analysis

To ensure that the data represents the national population, all analyses employed sampling weights. The weight variable used in our study was the 2-day dietary sample weight (WTDR2D), and we calculated new weights for the 2011–2018 period as 1/4 × WTDR2D. Continuous variables are presented as means ± standard deviation (SD), while categorical variables are presented as frequencies (percentages). Weighted *t*-tests were used to compare continuous variables, and weighted chi-square tests were employed for categorical variables. The relationship between DI-GM and sarcopenia was examined using weighted logistic regression. Three logistic regression models were established: Model 1 was unadjusted for potential confounding factors; Model 2 adjusted for covariates including age, sex, education level, marital status, PIR, and race; and Model 3 further adjusted for smoking, alcohol consumption, physical activity, hypertension, diabetes, and hyperlipidemia based on Model 2. Additionally, in Model 3, DI-GM was treated as a continuous variable, and restricted cubic splines (RCS) were used to illustrate the linear or non-linear association between DI-GM and Sarcopenia. RCS is a flexible regression method that allows for modeling curved trends without assuming a strict linear relationship, providing a more precise representation of the association. Compared to traditional linear regression, RCS can detect threshold effects or dose-response relationships, making the results more biologically meaningful. Subsequently, a stratified subgroup analysis was conducted based on model 3 to examine covariates. Interaction analyses were then performed to explore potential variations in associations among subgroups.

Based on the premises that “DI-GM is statistically significantly associated with DII” and “DII is statistically significantly associated with sarcopenia,” a mediation analysis was conducted to investigate whether the effect of DI-GM on sarcopenia is mediated by DII. The mediation effect was calculated using the “mediation” package in R software (23). Data processing was performed using R statistical software (version 4.3.1). Two-sided *p <* 0.05 were considered statistically significant.

## Results

### Baseline characteristics

This study included 9,470 participants aged 20 and older, inferring approximately 82.93 million U.S. adults. The prevalence of sarcopenia was found to be 7%, corresponding to around 6.18 million individuals. The average score of DI-GM was 5.00 (SD:1.54). In addition, DI-GM scores were lower in patients with sarcopenia compared to those without sarcopenia (5.00 vs. 5.30). Statistically significant differences (all *p* < 0.05) were observed between sarcopenic patients and non-sarcopenic individuals concerning age, race, education level, PIR, alcohol consumption, physical activity, hypertension, blood glucose levels, and hyperlipidemia. Patients with sarcopenia present as older, male, white, married, highly educated, and hyperlipidemic. Additionally, DII was higher in sarcopenic patients compared to non-sarcopenic individuals, also showing a statistically significant difference (*p* < 0.05). For more detailed information, refer to Table 1.

**TABLE 1:** Baseline characteristics of all participants were stratified by sarcopenia, weighted.

Characteristic	Overall, *N* = 82,937,614 (100%)	Non-sarcopenia, *N* = 76,753,310 (93%)	Sarcopenia, *N* = 6,184,304 (7%)	*P*-value
No. of participants in the sample	9,470	8,647	823	**–**
Age (%)				**<0.001**
20–30	23,927,466 (29%)	22,717,399 (30%)	1,210,066 (20%)	
31–40	19,353,342 (23%)	18,119,458 (24%)	1,233,885 (20%)	
>40	39,656,806 (48%)	35,916,453 (47%)	3,740,353 (60%)	
**Gender (%)**				0.068
Male	41,225,950 (50%)	37,873,237 (49%)	3,352,713 (54%)	
Female	41,711,664 (50%)	38,880,073 (51%)	2,831,591 (46%)	
Race (%)				**<0.001**
Non-Hispanic White	50,618,331 (61%)	47,607,114 (62%)	3,011,217 (49%)	
Other	13,952,928 (17%)	12,571,906 (16%)	1,381,022 (22%)	
Non-Hispanic Black	9,759,540 (12%)	9,523,861 (12%)	235,679 (3.8%)	
Mexican American	8,606,815 (10%)	7,050,430 (9.2%)	1,556,386 (25%)	
Married/live with partner (%)				0.512
No	32,019,117 (39%)	29,536,066 (38%)	2,483,051 (40%)	
Yes	50,918,497 (61%)	47,217,244 (62%)	3,701,253 (60%)	
Education level (%)				**<0.001**
Below high school	10,197,386 (12%)	8,777,997 (11%)	1,419,389 (23%)	
High school or above	72,740,228 (88%)	67,975,314 (89%)	4,764,915 (77%)	
PIR (%)				**<0.001**
Not poor	59,170,179 (76%)	55,393,294 (77%)	3,776,885 (66%)	
Poor	18,512,430 (24%)	16,562,508 (23%)	1,949,921 (34%)	
Smoking (%)				0.139
Never	49,266,968 (59%)	45,532,449 (59%)	3,734,519 (60%)	
Former	15,949,478 (19%)	14,605,362 (19%)	1,344,116 (22%)	
Current	17,721,168 (22%)	16,615,500 (22%)	1,105,669 (18%)	
Drinking (%)				<0.001
Former	6,397,294 (8%)	5,669,197 (8%)	728,097 (13%)	
Heavy	20,930,524 (27%)	19,323,929 (27%)	1,606,596 (28%)	
Mild	26,694,239 (34%)	25,284,773 (35%)	1,409,467 (25%)	
Moderate	16,190,944 (21%)	15,323,883 (21%)	867,062 (15%)	
Never	7,837,504 (10%)	6,730,402 (9%)	1,107,103 (19%)	
Physical activity (%)				**0.027**
Inactive	9,884,063 (14%)	9,056,872 (14%)	827,190 (19%)	
Active	59,188,963 (86%)	55,620,009 (86%)	3,568,954 (81%)	
Hypertension (%)				**<0.001**
No	60,707,731 (73%)	57,150,838 (74%)	3,556,893 (58%)	
Yes	22,229,883 (27%)	19,602,472 (26%)	2,627,410 (42%)	
Diabetes (%)				**<0.001**
No	75,742,773 (91%)	70,789,286 (92%)	4,953,486 (80%)	
Yes	7,194,841 (9%)	5,964,024 (8%)	1,230,818 (20%)	
Hyperlipidemia (%)				**<0.001**
No	32,281,732 (39%)	30,653,273 (40%)	1,628,459 (26%)	
Yes	50,655,882 (61%)	46,100,037 (60%)	4,555,844 (74%)	
DI_GM [mean (SD)]	5.27 (1.63)	5.30 (1.63)	5.00 (1.54)	**<0.001**
DI_GM, Tertile (%)				**0.011**
T1	10,574,292 (13%)	9,553,716 (12%)	1,020,576 (17%)	
T2	38,715,856 (47%)	35,640,205 (46%)	3,075,651 (50%)	
T3	33,647,466 (40%)	31,559,389 (42%)	2,088,077 (33%)	
DII [mean (SD)]	1.23 (1.86)	1.19 (1.87)	1.77 (1.69)	**<0.001**
DII, tertile (%)				**<0.001**
T1	27,634,608 (34%)	26,280,220 (34%)	1,354,388 (22%)	
T2	27,648,172 (33%)	25,467,579 (33%)	2,180,593 (35%)	
T3	27,654,834 (33%)	25,005,511 (33%)	2,649,322 (43%)	

The bold values are less than 0.05. Mean (SD) for continuous variables: the *P*-value was calculated by the weighted Student’s *t*-test. Percentages (weighted N, %) for categorical variables: the *P*-value was calculated by the weighted chi-square test. DI-GM, Dietary Index for Gut Microbiota; DII, Dietary Inflammatory Index; PIR, Ratio of family income to poverty.

### The association between DI-GM, DII, and sarcopenia

As shown in Table 2, three different logistic regression models were employed to evaluate the association between DI-GM scores and the prevalence of sarcopenia, all indicating a negative correlation (*p* < 0.001). In Model 3, after adjusting for various covariates, a one standard deviation increase in the DI-GM score was associated with a 15% reduction in the prevalence of sarcopenia [odds ratio (OR): 0.85 (95% confidence interval: 0.77, 0.94)]. Furthermore, when DI-GM was categorized into tertiles, the group with the highest DI-GM scores (T3) showed a 51% lower prevalence of sarcopenia compared to the group with the lowest scores (T1) [OR: 0.49 (95% confidence interval: 0.31, 0.79)].

**TABLE 2:** Association between DI-GM, DII, and sarcopenia, NHANES 2011–2018.

Characteristics	Model 1[OR (95% CI)]	*P*-value	Model 2[OR (95% CI)]	*P*-value	Model 3[OR (95% CI)]	*P*-value	Model 4[OR (95% CI)]	*P*-value
**DI-GM - sarcopenia**								
Continuous	0.89(0.84,0.94)	<0.001	0.87(0.82,0.92)	<0.001	0.85(0.77,0.94)	<0.001	0.86(0.78,0.94)	0.002
**Tertile**								
T1	1 (ref.)		1 (ref.)		1 (ref.)		1 (ref.)	
T2	0.81(0.59,1.11)	0.180	0.75(0.52,1.07)	0.110	0.74(0.48,1.14)	0.180	0.75(0.49,1.15)	0.180
T3	0.62(0.47,0.82)	0.001	0.56(0.41,0.76)	<0.001	0.49(0.31,0.79)	0.001	0.49(0.30,0.79)	0.004
P for trend	<0.001		<0.001		0.002		0.005	
**DII—sarcopenia**								
Continuous	1.20(1.12,1.28)	<0.001	1.23(1.15,1.33)	<0.001	1.28(1.17,1.41)	<0.001	1.26(1.12,1.42)	<0.001
**Tertile**								
T1	1 (ref.)		1 (ref.)		1 (ref.)		1 (ref.)	
T2	1.66(1.27,2.17)	<0.001	1.84(1.37,2.46)	<0.001	2.04(1.45,2.87)	<0.001	1.90(1.28,2.81)	0.002
T3	2.06(1.52,2.78)	<0.001	2.37(1.71,3.28)	<0.001	2.83(1.83,4.37)	<0.001	2.45(1.44,4.18)	0.002
P for trend	<0.001		<0.001		<0.001		0.003	

Model 1: no covariates were adjusted. Model 2: age, gender, education level, marital, PIR, and race were adjusted. Model 3: age, gender, education level, marital, PIR, race, smoking, drinking, physical activity, hypertension, diabetes, and hyperlipidemia were adjusted. Model 4: age, gender, education level, marital, PIR, race, smoking, drinking, physical activity, hypertension, diabetes, hyperlipidemia, protein intake, and caloric intake were adjusted. DI-GM, Dietary Index for Gut Microbiota; DII, Dietary Inflammatory Index; PIR, Ratio of family income to poverty; OR, odds ratio; CI, confidence interval.

**FIGURE 1:**
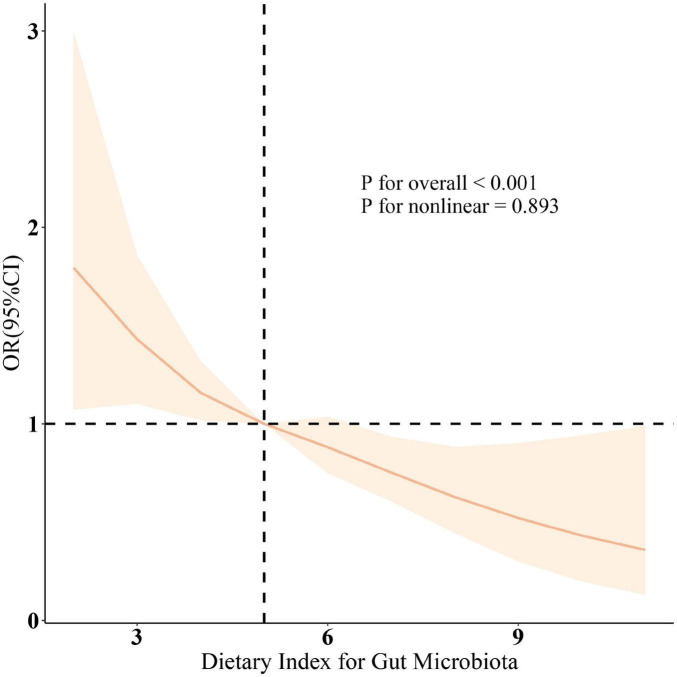
The restricted cubic spline (RCS) analysis between DI-GM and sarcopenia. OR (solid lines) and 95% confidence levels (shaded areas) were adjusted for age, gender, education level, marital, PIR, race, smoking, drinking, physical activity, hypertension, diabetes, and hyperlipidemia. RCS was used to illustrate the linear or nonlinear association between DI-GM and Sarcopenia. RCS is a flexible regression method that allows for modeling curved trends without assuming a strict linear relationship, providing a more precise representation of the association.

Additionally, the relationship between DII and sarcopenia was assessed, revealing a positive correlation across all three models (all *p* < 0.001). Higher DII scores were associated with an increased prevalence of sarcopenia, and the results were statistically significant (*p* < 0.05). Since dietary factors influence sarcopenia, we further included protein intake and total caloric intake in Model 4. The results remained robust. As shown in the RCS results (Figure 1), after adjusting for relevant variables, a significant negative correlation between DI-GM scores and the prevalence of sarcopenia was further confirmed (overall *P* < 0.001; non-linear *P* = 0.893).

### Subgroup analysis

**FIGURE 2:**
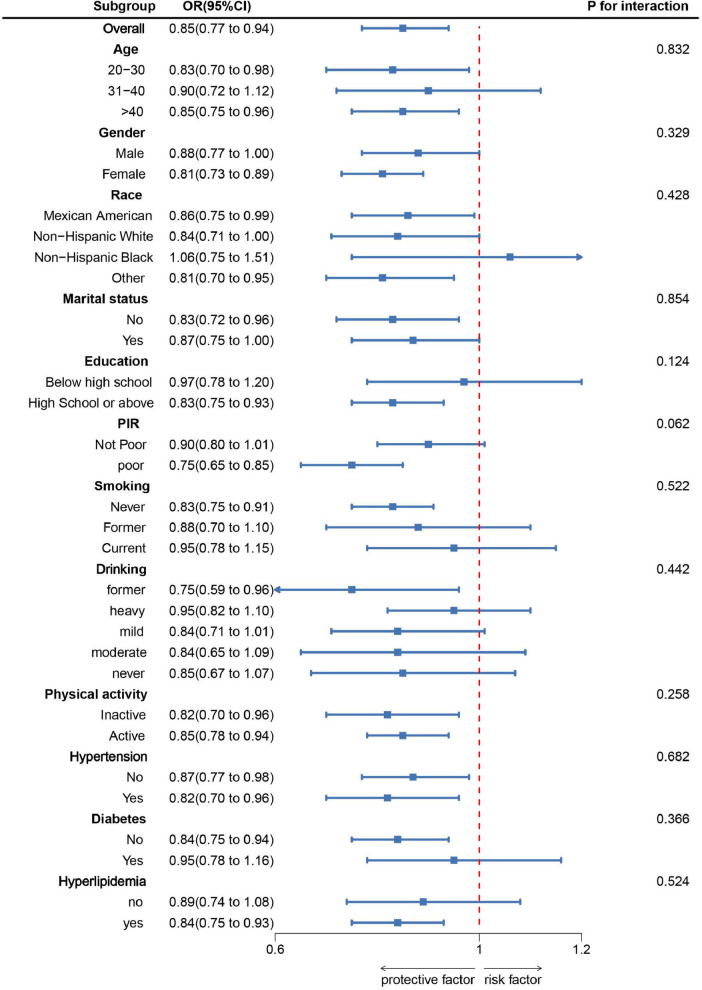
Subgroup analysis between DI-GM and Sarcopenia. ORs were calculated for each 1-unit increase in DI-GM. Analyses were adjusted for age, gender, education level, marital, PIR, race, smoking, drinking, physical activity, hypertension, diabetes, and hyperlipidemia.

As shown in Figure 2, subgroup analysis was conducted based on age, sex, race, marital status, education level, PIR, smoking, alcohol consumption, physical activity, hypertension, diabetes, and hyperlipidemia. The results indicated a negative correlation between DI-GM scores and sarcopenia prevalence in most subgroups. After adjusting for various confounding factors, no significant interaction effects were observed, suggesting that the association between DI-GM and sarcopenia is stable across different demographic and health-related factors. Specifically, a higher DI-GM was associated with a lower risk of sarcopenia (OR = 0.85, 95% CI: 0.77–0.94). The protective effect was significant in individuals aged 20–30 years (OR = 0.83, 95% CI: 0.70–0.98) and > 40 years (OR = 0.85, 95% CI: 0.75–0.96). The association was stronger in females (OR = 0.81, 95% CI: 0.73–0.89) than in males. Low-income individuals (OR = 0.75, 95% CI: 0.65–0.85) benefited more than non-poor individuals. Never-smokers and former drinkers had a stronger protective effect. DI-GM remained significant in individuals with hypertension and hyperlipidemia but not in those with diabetes.

### Mediation effect

**FIGURE 3:**
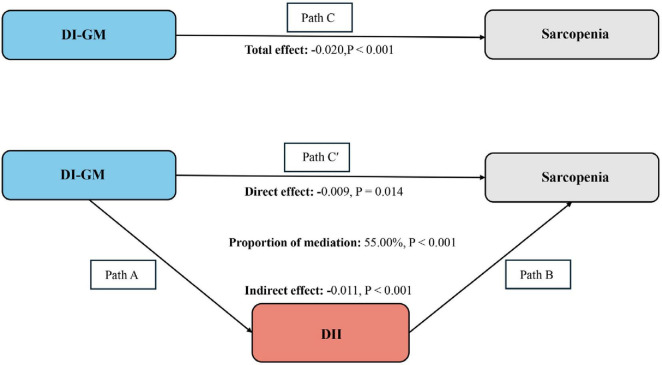
Schematic diagram of the mediation effect analysis. Path C indicates the total effect; path C’ indicates the direct effect. The indirect effect is estimated as the multiplication of paths A and B (path A*B). The mediated proportion is calculated as indirect effect/ (indirect effect + direct effect) × 100%. DI-GM, Dietary Index for Gut Microbiota; DII, Dietary Inflammatory Index. Analyses were adjusted for age, gender, education level, marital, PIR, race, smoking, drinking, physical activity, hypertension, diabetes, and hyperlipidemia. Mediation analysis was conducted to investigate whether the effect of DI-GM on sarcopenia is mediated by DII. The mediation effect was calculated using the “mediation” package in R software.

The mediation model and pathways are illustrated in Figure 3, with DI-GM as the independent variable, sarcopenia as the dependent variable, and DII as the mediating variable. As shown in Table 3, a significant correlation was observed between DI-GM and DII after adjusting for other covariates (β = −0.46, 95% CI: −0.49, −0.43). After adjusting for all covariates, the mediating effect of DII was identified (Figure 3) (indirect effect = −0.011, P < 0.001; direct effect = −0.009, *P* = 0.014). Therefore, DII can be considered a mediating factor in the association between DI-GM and the likelihood of sarcopenia.

**TABLE 3:** Multivariate linear regression of DI-GM and DII.

	β	95%CI	*P*-value
DI-GM-DII	−0.46	(−0.49, −0.43)	<0.001

Adjusted for age, gender, education level, marital, PIR, race, smoking, drinking, physical activity, hypertension, diabetes, and hyperlipidemia.

## Discussion

This cross-sectional analysis reveals a negative correlation between DI-GM and the prevalence of sarcopenia, while DII shows a positive correlation with sarcopenia prevalence. To our knowledge, this is the first cross-sectional study to explore the relationship between DI-GM and sarcopenia. Additionally, mediation analysis indicates that DII mediates the relationship between DI-GM and sarcopenia. As the understanding of the connections between diet and inflammation, as well as inflammation and health, advances, DII can be utilized to assess the impact of diet on health outcomes (24). Therefore, dietary management for sarcopenia holds significant implications for clinical practitioners. This study suggests that adherence to a DI-GM dietary pattern may influence the development of sarcopenia by reducing the inflammatory potential of the diet.

Changes in the composition of the gut microbiota may actually promote chronic inflammation and metabolic resistance, ultimately leading to reduced muscle size, impaired muscle function, and adverse clinical outcomes (25). Compared to existing dietary indices, the Dietary Gut Microbiota Index (DI-GM) can effectively identify dietary patterns that are beneficial or harmful to gut microbiota, potentially serving as a standardized tool for assessing a balanced diet for gut health (26). One unique aspect of DI-GM is the inclusion of fermented dairy products, which can modulate gut microbiota. The metabolites produced by the gut microbiota are believed to have potential effects on skeletal muscle, potentially through mechanisms such as the action of short-chain fatty acids (SCFAs). SCFAs, particularly butyrate, propionate, and acetate, exert anti-inflammatory effects by inhibiting histone deacetylases (HDACs) and activating GPR41/GPR43, reducing pro-inflammatory cytokines (27). Studies have shown that SCFAs enhance muscle mass and function by activating the mTOR signaling pathway in muscle-reducing mice to mitigate age-related muscle loss and dysfunction (28). SCFAs also inhibit the production of inflammatory markers via the NF-κB pathway (29), and chronic inflammation is a hallmark of skeletal muscle disorders (30). Furthermore, dysbiosis may reduce secondary bile acids, which are associated with a nuclear receptor (FXR). Animal studies have indicated that bile acids, FXR signaling, and the expression of FGF15/19 (fibroblast growth factors 15/19) may influence host skeletal muscle metabolism by promoting muscle growth and mitigating muscle atrophy (8, 31, 32). Additionally, one of the metabolites, branched-chain amino acids (BCAAs), enhances insulin-mediated glucose metabolism in liver and muscle cells, which may also be a potential mechanism by which gut microbiota influence skeletal muscle (33). A study suggested that the downregulation of certain key species in the gut of sarcopenia patients may affect muscle mass and physical function through the combined effects of various metabolites (34). Another cohort study indicated that reduced gut microbial diversity in men is associated with a low skeletal muscle mass index (SMI) (35), which aligns with our findings on the association between DI-GM and sarcopenia.

Chronic low-grade inflammation is known to be associated with the pathogenesis of several chronic non-communicable diseases (13). A low dietary quality score is often correlated with elevated levels of plasma IL-6, E-selectin, and soluble ICAM-1 (36). Furthermore, a randomized controlled trial demonstrated that the intake of fermented foods increased the diversity of the microbiome while reducing several pro-inflammatory cytokines and chemokines (37). Anti-inflammatory dietary patterns can decrease inflammation and thus prevent muscle loss (38). The Dietary Inflammatory Index (DII), which is based on six inflammatory biomarkers, effectively reflects the relationship between diet and inflammation (12). Several studies have indicated a correlation between DII and muscle mass (14, 15). The relationship between the inflammatory index and skeletal muscle mass may be attributed to inflammatory factors (including CRP, IL-6, and TNF-α) that inhibit the activity of the insulin-like growth factor 1 (IGF-1), activating the ubiquitin-proteasome system and leading to metabolic resistance and the loss of muscle homeostasis (39). Moreover, inflammation is associated with early malnutrition. In experimental models, the abundance of key taxa related to inflammation regulation (such as *Escherichia coli*) has been positively correlated with satiety and satiety hormone levels, suggesting that the microbiome may mediate muscle wasting by promoting malnutrition through inflammation (25, 40). On the other hand, a healthy gut microbiome regulates immune homeostasis, maintaining a balance between pro-inflammatory and anti-inflammatory responses while enhancing barrier function (32). Consequently, dysbiosis and changes in the gut barrier can lead to increased absorption of lipopolysaccharides (LPS), termed metabolic endotoxemia. LPS significantly increases NF-κB binding activity in skeletal muscle and JNK phosphorylation, collectively suppressing insulin signaling (41). Animal experiments have also shown that LPS injection induces various changes, including a reduced abundance of Bacteroidetes, increased serum concentrations of pro-inflammatory cytokines, and impaired mitochondrial morphology (41). The immune stress mediated by these changes can adversely affect muscle growth in weaned piglets (32, 42). Additionally, another study indicated that the administration of TAK-242, a Toll-like receptor 4 (TLR4) specific signaling inhibitor, reversed muscle atrophy induced by endotoxemia in mice (43). Understanding how gut microbiota interacts with host muscle provides a foundation for therapeutic microbial interventions aimed at combating or preventing disease.

This study provides further insights into the potential mechanisms by which dietary patterns influence the development of sarcopenia. Specifically, diets with high DII scores, such as those rich in red and processed meats, high-fat, and high-sugar foods, are closely associated with pro-inflammatory states (44, 45). In contrast, diets with low DII scores, including those rich in fruits, vegetables, and nuts, exhibit anti-inflammatory properties (44, 45). Additionally, diets with high DI-GM scores, characterized by foods rich in dietary fiber and beneficial components (e.g., whole grains, legumes, vegetables, and fermented dairy) (11), can modulate gut microbiota composition and function, indirectly influencing inflammation levels. Diets high in dietary fiber and beneficial components serve as substrates for gut microbiota fermentation, leading to the production of short-chain fatty acids (SCFAs), such as butyrate, acetate, and propionate (46). These SCFAs exert anti-inflammatory effects by modulating immune cell activity (47). These dietary patterns may play a protective role against sarcopenia by reducing inflammation and improving gut microbiota health. Based on these findings, we recommend adopting anti-inflammatory and prebiotic-rich dietary patterns, such as the Mediterranean diet (48) and the DASH diet (49), as potential strategies for sarcopenia prevention and treatment. Combining these dietary interventions with protein supplementation and resistance training (50, 51)may further enhance their effectiveness. Furthermore, this study provides new directions for future research. Prospective studies are needed to validate the causal relationships between DI-GM, DII, and sarcopenia. Animal experiments could further explore the gut microbiota-inflammation-muscle metabolism axis to provide direct evidence for the underlying mechanisms. Additionally, future research could investigate personalized dietary intervention strategies based on DI-GM and DII to evaluate their practical applications in sarcopenia prevention and treatment. These studies will help optimize dietary strategies for sarcopenia management and provide a scientific basis for public health policies. Therefore, our study indicates that there are associations among gut microbiota, diet, and sarcopenia. Given that multiple studies have linked gut microbiota to inflammatory bowel disease, epilepsy, and cancer (52–54), this research provides new insights into the prevention and management of sarcopenia through dietary management aimed at modulating gut microbiota. This study found that DI-GM was negatively associated with sarcopenia prevalence (OR = 0.85, 95% CI: 0.77, 0.94), while DII was positively associated with sarcopenia prevalence (OR = 1.28, 95% CI: 1.17, 1.41). These findings further support the aforementioned hypothesis.

Our study has several advantages. First, it is based on the NHANES database, which provides a large sample size and allows for the analysis of confounding factors, subgroup analysis, and mediation analysis, yielding stable results. This study demonstrates a negative correlation between DI-GM and the prevalence of sarcopenia, suggesting that DII may mediate this relationship. Second, as a newly proposed Dietary Index, DI-GM has the potential to serve as a standardized tool for assessing balanced diets that support gut microbiota. Its visual approach to evaluating the relationship between diet and sarcopenia offers a more convenient and implementable solution for clinical guidance. Additionally, we conducted a mediation analysis to explore the relationships among DII, DI-GM, and sarcopenia.

Despite the significant contributions of this study, several limitations should be noted: (1) As a cross-sectional study, it cannot establish a causal relationship between DI-GM and the prevalence of sarcopenia. Further prospective studies with larger and more diverse samples are needed to elucidate the causal relationship between DI-GM and sarcopenia. (2) The data for this study were sourced from the NHANES dataset covering 2011–2018, which means that the results apply only to U.S. adults and may have limited applicability to populations in other countries. (3) The dietary data in this study relied on questionnaires recorded by NHANES, and participants’ responses may be subject to recall bias. (4) Although this study has adjusted for many potential confounding factors, it is still unable to exclude the possibility of confounding effects from unmeasured or unidentified factors.

## Conclusion

In conclusion, our study enhances the understanding of the association between DI-GM dietary patterns and sarcopenia. This knowledge is beneficial for future dietary recommendations aimed at preventing diseases related to skeletal muscle health. By tracking an individual’s dietary habits, the use of DI-GM scoring can help assess the health impacts of diet on the gut microbiota. This approach is particularly valuable for clinical nutritionists, public health practitioners, and researchers, providing important strategies for the clinical prevention and management of sarcopenia. Finally, the mediation analysis involving DII indicates that an anti-inflammatory diet can contribute to the reduction of sarcopenia prevalence through its effects on DI-GM.

## Data Availability

The datasets presented in this study can be found in online repositories. The names of the repository/repositories and accession number(s) can be found at: https://wwwn.cdc.gov/nchs/nhanes/.

## References

[1] Cruz-Jentoft A Sayer A. Sarcopenia. *Lancet.* (2019) 393:2636–46. 10.1016/S0140-6736(19)31138-9 31171417

[2] Dennison E Sayer A Cooper C. Epidemiology of sarcopenia and insight into possible therapeutic targets. *Nat Rev Rheumatol.* (2017) 13:340–7. 10.1038/nrrheum.2017.60 28469267 PMC5444517

[3] Liu C Cheung W Li J Chow S Yu J Wong S Understanding the gut microbiota and sarcopenia: A systematic review. *J Cachexia Sarcopenia Muscle.* (2021) 12:1393. 10.1002/jcsm.12784 34523250 PMC8718038

[4] Yuan S Larsson S. Epidemiology of sarcopenia: Prevalence, risk factors, and consequences. *Metabolism.* (2023) 144:155533. 10.1016/j.metabol.2023.155533 36907247

[5] Huang Q Wan J Nan W Li S He B Peng Z. Association between manganese exposure in heavy metals mixtures and the prevalence of sarcopenia in US adults from NHANES 2011-2018. *J Hazard Mater.* (2024) 464:133005. 10.1016/j.jhazmat.2023.133005 37988867

[6] Bowyer R Jackson M Pallister T Skinner J Spector T Welch A Use of dietary indices to control for diet in human gut microbiota studies. *Microbiome.* (2018) 6:77. 10.1186/s40168-018-0455-y 29695307 PMC5918560

[7] Zmora N Suez J Elinav E. You are what you eat: Diet, health and the gut microbiota. *Nat Rev Gastroenterol Hepatol.* (2019) 16:35–56. 10.1038/s41575-018-0061-2 30262901

[8] Mancin L Wu G Paoli A. Gut microbiota-bile acid-skeletal muscle axis. *Trends Microbiol.* (2023) 31:254–69. 10.1016/j.tim.2022.10.003 36319506

[9] Chen L Chang S Chang H Wu C Pan C Chang C Probiotic supplementation attenuates age-related sarcopenia via the gut-muscle axis in SAMP8 mice. *J Cachexia Sarcopenia Muscle.* (2022) 13:515–31. 10.1002/jcsm.12849 34766473 PMC8818665

[10] Zhao J Liang R Song Q Song S Yue J Wu C. Investigating association between gut microbiota and sarcopenia-related traits: A Mendelian randomization study. *Precis Clin Med.* (2023) 6:bad010. 10.1093/pcmedi/pbad010 37324750 PMC10263384

[11] Kase B Liese A Zhang J Murphy E Zhao L Steck S. The development and evaluation of a literature-based dietary index for gut microbiota. *Nutrients.* (2024) 16:1045. 10.3390/nu16071045 38613077 PMC11013161

[12] Shivappa N Steck S Hurley T Hussey J Hébert J. Designing and developing a literature-derived, population-based dietary inflammatory index. *Public Health Nutr.* (2014) 17:1689–96. 10.1017/S1368980013002115 23941862 PMC3925198

[13] Marx W Veronese N Kelly J Smith L Hockey M Collins S The Dietary inflammatory index and human health: An umbrella review of meta-analyses of observational studies. *Adv Nutr.* (2021) 12:1681–90. 10.1093/advances/nmab037 33873204 PMC8483957

[14] Khamoushi F Soleimani D Najafi F Ahmadi N Heidarzadeh-Esfahani N Anvari B Association between dietary inflammatory index and musculoskeletal disorders in adults. *Sci Rep.* (2023) 13:20302. 10.1038/s41598-023-46429-w 37985726 PMC10662012

[15] Su Y Yeung S Chen Y Leung J Kwok T. The associations of dietary inflammatory potential with musculoskeletal health in Chinese community-dwelling older people: The Mr/Ms. OS (Hong Kong) cohort study. *J Bone Miner Res Off J Am Soc Bone Miner Res.* (2022) 37:1179. 10.1002/jbmr.4556 35416312 PMC9177744

[16] Nardone O de Sire R Petito V Testa A Villani G Scaldaferri F Inflammatory bowel diseases and sarcopenia: The role of inflammation and gut microbiota in the development of muscle failure. *Front Immunol.* (2021) 12:694217. 10.3389/fimmu.2021.694217 34326845 PMC8313891

[17] Liu D Wang S Liu S Wang Q Che X Wu G. Frontiers in sarcopenia: Advancements in diagnostics, molecular mechanisms, and therapeutic strategies. *Mol Aspects Med.* (2024) 97:101270. 10.1016/j.mam.2024.101270 38583268

[18] Diao H Yan F He Q Li M Zheng Q Zhu Q Association between dietary inflammatory index and sarcopenia: A meta-analysis. *Nutrients.* (2023) 15:219. 10.3390/nu15010219 36615879 PMC9824141

[19] Zhou H Su H Gong Y Chen L Xu L Chen G The association between weight-adjusted-waist index and sarcopenia in adults: A population-based study. *Sci Rep.* (2024) 14:10943. 10.1038/s41598-024-61928-0 38740910 PMC11091224

[20] Xu W Mu D Wang Y Wang Y Wang C Zhang X. Association between oxidative balance score and sarcopenia in US adults: NHANES 2011–2018. *Front Nutr.* (2024) 11:1342113. 10.3389/fnut.2024.1342113 38721026 PMC11076835

[21] Zuercher M Harvey D Au L Shadyab A Santiago-Torres M Liu S Energy-adjusted dietary inflammatory index and diabetes risk in postmenopausal hispanic women. *J Acad Nutr Diet.* (2024) 124:1431–9. 10.1016/j.jand.2023.08.002 37544374 PMC10839112

[22] Qing L Zhu Y Yu C Zhang Y Ni J. Exploring the association between dietary inflammatory index and chronic pain in US adults using NHANES 1999-2004. *Sci Rep.* (2024) 14:8726. 10.1038/s41598-024-58030-w 38622145 PMC11018766

[23] Huang S He Q Wang X Choi S Gong H. Associations of the planetary health diet index (PHDI) with asthma: The mediating role of body mass index. *BMC Public Health.* (2024) 24:2305. 10.1186/s12889-024-19856-1 39187832 PMC11346270

[24] Hébert J Shivappa N Wirth M Hussey J Hurley T. Perspective: The dietary inflammatory index (DII)-lessons learned, improvements made, and future directions. *Adv Nutr.* (2019) 10:185–95. 10.1093/advances/nmy071 30615051 PMC6416047

[25] Ticinesi A Nouvenne A Cerundolo N Catania P Prati B Tana C Gut microbiota, muscle mass and function in aging: A focus on physical frailty and sarcopenia. *Nutrients.* (2019) 11:1633. 10.3390/nu11071633 31319564 PMC6683074

[26] Zhang X Yang Q Huang J Lin H Luo N Tang H. Association of the newly proposed dietary index for gut microbiota and depression: The mediation effect of phenotypic age and body mass index. *Eur Arch Psychiatry Clin Neurosci.* (2024): 10.1007/s00406-024-01912-x Online ahead of print. 39375215

[27] Vinolo M Rodrigues H Nachbar R Curi R. Regulation of inflammation by short chain fatty acids. *Nutrients.* (2011) 3:858–76. 10.3390/nu3100858 22254083 PMC3257741

[28] Liu C Wong P Wang Q Wong H Huang T Cui C Short-chain fatty acids enhance muscle mass and function through the activation of mTOR signalling pathways in sarcopenic mice. *J Cachexia Sarcopenia Muscle.* (2024) 15:2387–401. 10.1002/jcsm.13573 39482890 PMC11634463

[29] Boulangé C Neves A Chilloux J Nicholson J Dumas M. Impact of the gut microbiota on inflammation, obesity, and metabolic disease. *Genome Med.* (2016) 8:42. 10.1186/s13073-016-0303-2 27098727 PMC4839080

[30] Wu K Shieh J Qin L Guo J. Mitochondrial mechanisms in the pathogenesis of chronic inflammatory musculoskeletal disorders. *Cell Biosci.* (2024) 14:76. 10.1186/s13578-024-01259-9 38849951 PMC11162051

[31] Aslam H Marx W Rocks T Loughman A Chandrasekaran V Ruusunen A The effects of dairy and dairy derivatives on the gut microbiota: A systematic literature review. *Gut Microbes.* (2020) 12:1799533. 10.1080/19490976.2020.1799533 32835617 PMC7524346

[32] Li T Yin D Shi R. Gut-muscle axis mechanism of exercise prevention of sarcopenia. *Front Nutr.* (2024) 11:1418778. 10.3389/fnut.2024.1418778 39221163 PMC11362084

[33] Daniel N Nachbar R Tran T Ouellette A Varin T Cotillard A Gut microbiota and fermentation-derived branched chain hydroxy acids mediate health benefits of yogurt consumption in obese mice. *Nat Commun.* (2022) 13:1343. 10.1038/s41467-022-29005-0 35292630 PMC8924213

[34] He Y Cui W Fang T Zhang Z Zeng M. Metabolites of the gut microbiota may serve as precise diagnostic markers for sarcopenia in the elderly. *Front Microbiol.* (2023) 14:1301805. 10.3389/fmicb.2023.1301805 38188577 PMC10768011

[35] Park C Lee E Kim H Lee Y Yoon K Kim H. Sex-specific associations between gut microbiota and skeletal muscle mass in a population-based study. *J Cachexia Sarcopenia Muscle.* (2022) 13:2908–19. 10.1002/jcsm.13096 36218092 PMC9745450

[36] Gill P Inniss S Kumagai T Rahman F Smith A. The role of diet and gut microbiota in regulating gastrointestinal and inflammatory disease. *Front Immunol.* (2022) 13:866059. 10.3389/fimmu.2022.866059 35450067 PMC9016115

[37] Armet A Deehan E O’Sullivan A Mota J Field C Prado C Rethinking healthy eating in light of the gut microbiome. *Cell Host Microbe.* (2022) 30:764–85. 10.1016/j.chom.2022.04.016 35679823

[38] Ker A Kao P. Methodological considerations on the association between dietary inflammatory potential and musculoskeletal health. *J Bone Miner Res.* (2022) 37:2678–9. 10.1002/jbmr.4635 35775244

[39] Xie H Wang H Wu Z Li W Liu Y Wang N. The association of dietary inflammatory potential with skeletal muscle strength, mass, and sarcopenia: A meta-analysis. *Front Nutr.* (2023) 10:1100918. 10.3389/fnut.2023.1100918 37255936 PMC10225560

[40] Wells J Sawaya A Wibaek R Mwangome M Poullas M Yajnik C The double burden of malnutrition: Aetiological pathways and consequences for health. *Lancet.* (2020) 395:75–88. 10.1016/S0140-6736(19)32472-9 31852605 PMC7613491

[41] Grosicki G Fielding R Lustgarten M. Gut microbiota contribute to age-related changes in skeletal muscle size, composition, and function: Biological basis for a gut-muscle axis. *Calcif Tissue Int.* (2018) 102:433–42. 10.1007/s00223-017-0345-5 29058056 PMC5858871

[42] Yu J Zheng C Guo Q Yin Y Duan Y Li F. LPS-related muscle loss is associated with the alteration of Bacteroidetes abundance, systemic inflammation, and mitochondrial morphology in a weaned piglet model. *Sci China Life Sci.* (2024) 67:1970–88. 10.1007/s11427-023-2552-7 38913237

[43] Ono Y Maejima Y Saito M Sakamoto K Horita S Shimomura K TAK-242, a specific inhibitor of Toll-like receptor 4 signalling, prevents endotoxemia-induced skeletal muscle wasting in mice. *Sci Rep.* (2020) 10:694. 10.1038/s41598-020-57714-3 31959927 PMC6970997

[44] Szypowska A Regulska-Ilow B Zatońska K Szuba A. Comparison of intake of food groups based on dietary inflammatory index (DII) and cardiovascular risk factors in the middle-age population of lower Silesia: Results of the PURE poland study. *Antioxidants.* (2023) 12:285. 10.3390/antiox12020285 36829844 PMC9952843

[45] Szypowska A Zatońska K Szuba A Regulska-Ilow B. Dietary inflammatory index (DII)^®^ and metabolic syndrome in the selected population of polish adults: Results of the PURE poland sub-study. *Int J Environ Res Public Health.* (2023) 20:1056. 10.3390/ijerph20021056 36673811 PMC9859570

[46] Liu X Shao J Liao Y Wang L Jia Y Dong P Regulation of short-chain fatty acids in the immune system. *Front Immunol.* (2023) 14:1186892. 10.3389/fimmu.2023.1186892 37215145 PMC10196242

[47] Portincasa P Bonfrate L Vacca M De Angelis M Farella I Lanza E Gut microbiota and short chain fatty acids: Implications in glucose homeostasis. *Int J Mol Sci.* (2022) 23:1105. 10.3390/ijms23031105 35163038 PMC8835596

[48] Casas R Sacanella E Estruch R. The immune protective effect of the Mediterranean diet against chronic low-grade inflammatory diseases. *Endocr Metab Immune Disord-Drug Targets.* (2014) 14:245–54. 10.2174/1871530314666140922153350 25244229 PMC4443792

[49] Soltani S Chitsazi M Salehi-Abargouei A. The effect of dietary approaches to stop hypertension (DASH) on serum inflammatory markers: A systematic review and meta-analysis of randomized trials. *Clin Nutr.* (2018) 37:542–50. 10.1016/j.clnu.2017.02.018 28302405

[50] Mende E Moeinnia N Schaller N Weiß M Haller B Halle M Progressive machine-based resistance training for prevention and treatment of sarcopenia in the oldest old: A systematic review and meta-analysis. *Exp Gerontol.* (2022) 163:111767. 10.1016/j.exger.2022.111767 35318104

[51] Vikberg S Sörlén N Brandén L Johansson J Nordström A Hult A Effects of resistance training on functional strength and muscle mass in 70-year-old individuals with pre-sarcopenia: A randomized controlled trial. *J Am Med Dir Assoc.* (2019) 20:28–34. 10.1016/j.jamda.2018.09.011 30414822

[52] Li W Deng Y Chu Q Zhang P. Gut microbiome and cancer immunotherapy. *Cancer Lett.* (2019) 447:41–7. 10.1016/j.canlet.2019.01.015 30684593

[53] Dahlin M Prast-Nielsen S. The gut microbiome and epilepsy. *EBioMedicine.* (2019) 44:741–6. 10.1016/j.ebiom.2019.05.024 31160269 PMC6604367

[54] Shan Y Lee M Chang E. The gut microbiome and inflammatory bowel diseases. *Annu Rev Med.* (2022) 73:455–68. 10.1146/annurev-med-042320-021020 34555295 PMC10012812

