# Supplementary material for: The association between Dietary Index for Gut Microbiota and sarcopenia: the mediating role of Dietary Inflammatory Index
Source: Front Nutr. 2025 Mar 31;12:1514209. doi: 10.3389/fnut.2025.1514209 (PMC11994312; doi:10.3389/fnut.2025.1514209)
Supplement: Supplementary file 1 [file Table_1.docx]

**Supplementary Material**

**Calculation of the dietary inflammatory index(DII)[1].**

Calculation of the DII is based on dietary intake data that are then linked to the regionally representative world database that provided a robust estimate of a mean and standard deviation for each parameter. These then become the multipliers to express an individual's exposure relative to the ‘standard global mean’ as a Z-score. This is achieved by subtracting the ‘standard mean’ from the amount reported and dividing this value by its standard deviation. To minimize the effect of ‘right skewing’, this value is converted to a percentile score. To achieve a symmetrical distribution with values centred on 0 (null) and bounded between −1 (maximally anti-inflammatory) and +1 (maximally pro-inflammatory), each percentile score is doubled and then ‘1’ is subtracted.

The centred percentile value for each food parameter is then multiplied by its respective ‘overall food parameter-specific inflammatory effect score’ to obtain the ‘food parameter-specific DII score’. Finally, all of the ‘food parameter-specific DII scores’ are summed to create the ‘overall DII score’ for an individual.

The 28 nutrients in this study were alcohol, β-carotene, caffeine, carbohydrate, cholesterol, energy, total fat, fiber, folic acid, iron, magnesium, monounsaturated fatty acids, polyunsaturated fatty acids, n-3 fatty acids, n-6 fatty acids, protein, saturated fat, selenium, zinc, vitamin A, B1, B2, B3, B6, B12, C, D, and E[2].

1. Shivappa N, Steck SE, Hurley TG, Hussey JR, Hébert JR. Designing and developing a literature-derived, population-based dietary inflammatory index. Public Health Nutrition. 2014;17:1689–96.

2. Meng X, Sha W, Lou X, Chen J. The relationship between dietary inflammatory index and osteoporosis among chronic kidney disease population. Sci Rep. 2023;13:22867.

**Table S1. Definition of components in DI-GM.**

| **Component** | **Included Foods within the Component** | **Scoring** |
| --- | --- | --- |
| **Beneficial to gut microbiota** |  |  |
| Avocados | Avocados | For each component, a score of 1 if consumption at or above the sex-specific median, else 0 |
| Broccoli | Broccoli |  |
| Chickpea | Chickpeas |  |
| Coffee | Coffee |  |
| Cranberries | Cranberries |  |
| Fermented dairy | Yogurt, cheese, kefir, sour cream, buttermilk |  |
| Fiber | Not applicable |  |
| Soybean | Soy products—Soy milk, Tofu |  |
| Whole grains | Grains defined as whole grains, containing the entire grain kernel―the bran, germ, and endosperm |  |
| **Unfavorable to gut microbiota** |  |  |
| High-fat diet (% energy) | Not applicable | 0 if consumption at or above 40% energy from fat, else 1 For each remaining component, a score of 0 if consumption at or above the sex-specific median, else 1 |
| Processed meat | Frankfurters, sausages, corned beef, and luncheon meat that are made from beef, pork, or poultry |  |
| Red meat | Beef, veal, pork, lamb, and game meat; excludes organ meat and cured meat |  |
| Refined grains | Refined grains that do not contain all of the components of the entire grain kernel |  |

**Table S2.** Description of covariates

| Covariates | Description in NHANES |
| --- | --- |
| Age | Divided into three groups: 20-40 years old, 41-60  years old, >60 years old |
| Gender | Male and Female |
| Race | Mexican American, Non-Hispanic Black, Non-Hispanic White, Other Race |
| Educational level | Below high school, High School or above |
| Marital status | Yes: Married/Living with partner |
| PIR | Poor: <1.3; Not Poor:>=1.3 |
| Physical activity | Active physical activity was defined as >599 MET, or >149 min of moderate physical activity, or >74 min of vigorous physical activity |
| Smoking | Smoking status was grouped into never smoker (defined as <100 cigarettes in a lifetime), current smoker (defined as ≥100 cigarettes in a lifetime), and former smoker (defined as ≥100 cigarettes and had quit smoking) |
| Drinking | heavy drinking (≥4 drinks/day for men, ≥3 drinks/day for women, or ≥5 days of drinking in a month),  moderate drinking (≥3 drinks/day for men, ≥2 drinks/day for women, or ≥2 days of drinking in a month),  mild drinking (≤2 drinks/day for men, ≤1 drink/day for women, and ≥12 drinks in a year),  and never-drinking (total number of drinks in a year <12, and dietary alcohol content of 0%) |
| Diabetes | Diabetes was defined as a history of previous diabetes, HbA1c level ≥6.5%, or fasting blood glucose level ≥126 mg/dL |
| Hypertension | The diagnostic criteria consist of self-reported hypertension history, the utilization of antihypertensive medication, a systolic blood pressure (SBP) ≥ 140mmHg, or a diastolic blood pressure (DBP) ≥ 90mmHg |
| Hyperlipidemia | (1) Triglyceride (TG) levels ≥150 mg/dl (1.7 mmol/L);(2) Total cholesterol (TC) levels ≥200 mg/dl (5.18 mmol/L);(3) Low-density lipoprotein (LDL) levels ≥130 mg/dl (3.37 mmol/L);(4) High-density lipoprotein (HDL) levels: Men: <40 mg/dl (1.04 mmol/L); Women: <50 mg/dl (1.30 mmol/L); (5) Individuals taking cholesterol-lowering drugs are also considered hyperlipidemia. |

PIR, Ratio of family income to poverty.


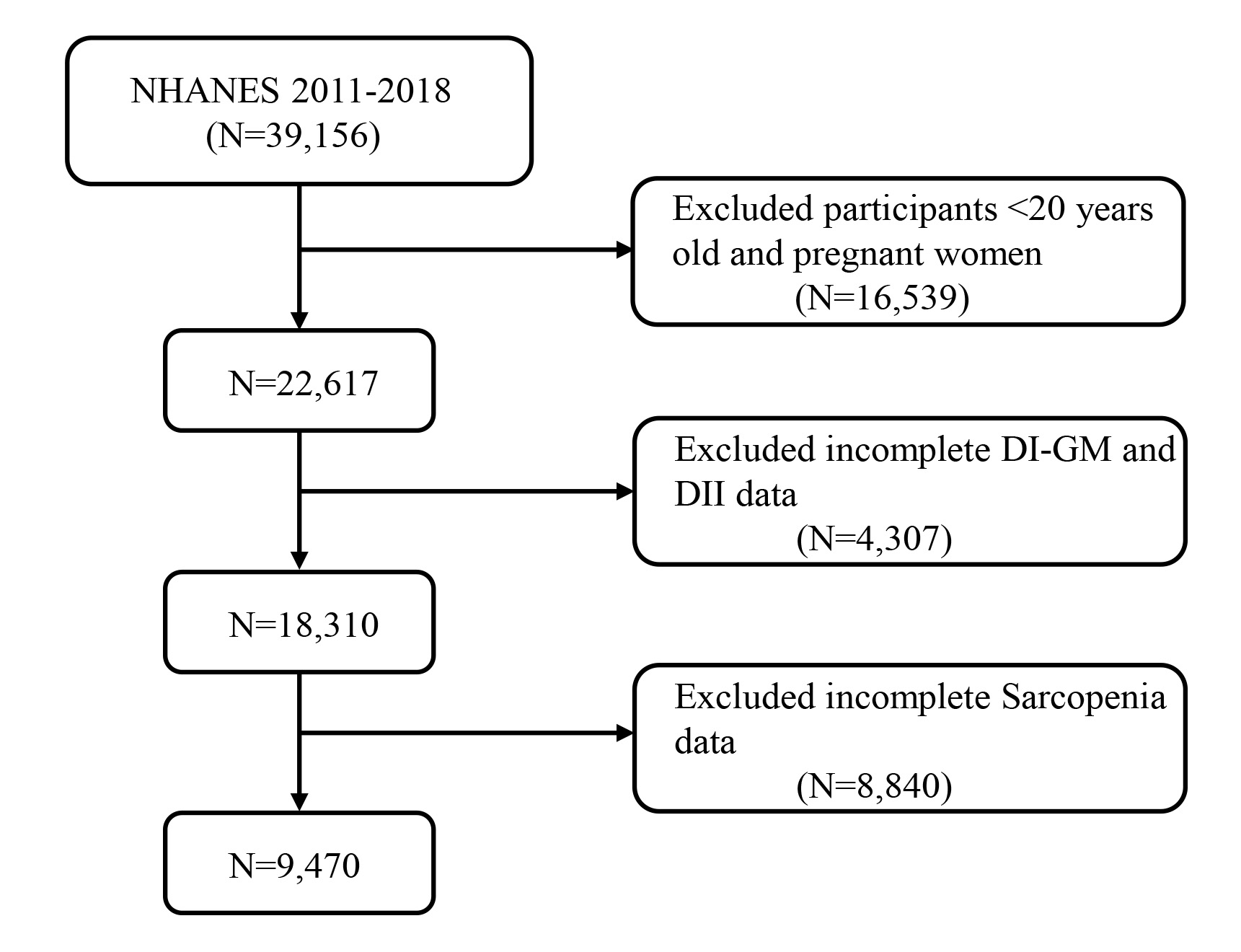


**Figure S1.** A flow diagram of eligible participant selection in the National Health and Nutrition Examination Survey.

Abbreviation: DI-GM, Dietary Index for Gut Microbiota; DII, Dietary Inflammatory Index.
